# Editorial: Classic and Pleiotropic Actions of Vitamin D

**DOI:** 10.3389/fendo.2019.00341

**Published:** 2019-05-29

**Authors:** Pawel Pludowski, William B. Grant, Jerzy Konstantynowicz, Michael F. Holick

**Affiliations:** ^1^Department of Biochemistry, Radioimmunology and Experimental Medicine, The Children's Memorial Health Institute, Warsaw, Poland; ^2^Sunlight, Nutrition, and Health Research Center, San Francisco, CA, United States; ^3^Department of Pediatric Rheumatology, Immunology, and Metabolic Bone Diseases, Medical University of Bialystok, Bialystok, Poland; ^4^Department of Medicine, Section of Endocrinology, Diabetes, and Nutrition, Boston University School of Medicine, Boston Medical Center, Boston, MA, United States

**Keywords:** vitamin D deficiency, vitamin D supplementation, 25(OH)D, pleiotropic action, vitamin D guidelines

Vitamin D deficiency is the world-wide health problem. Vitamin D deficiency has been associated with a wide variety of acute and chronic diseases including infectious diseases, autoimmune diseases including multiple sclerosis, type 1 diabetes and rheumatoid arthritis, cardiovascular heart disease and stroke, type 2 diabetes, depression and neurocognitive dysfunction, and several fatal malignancies and cancer (1, 2). It is also recognized that vitamin D deficiency is associated with increased mortality and negative birth outcomes (3–7). This recognition has not only led to a marked increase in the sales figures of supplements and prescriptions containing vitamin D, but has also resulted in innovative vitamin D food fortification programs, including those implemented most recently in India (8, 9). These continue to cause concerns about children and adults ingesting too much vitamin D that could potentially lead to toxicity including hypercalcemia, nephrocalcinosis, nephrolithiasis, or cardiovascular calcification.

The International “Vitamin D- minimum, maximum, optimum” (co-organized by the European Vitamin D Association, EVIDAS) has over the past several years served as a forum for scientists, clinicians and health care professionals to discuss various aspects of vitamin D related to health and disease. The 3rd International Conference “Vitamin D- minimum, maximum, optimum” (EVIDAS 2017) held in Warsaw (Poland) in September 22–23, 2017 (www.witaminad.waw.pl) was attended by scientists and health care professionals who discussed a broad spectrum of topics and controversies on vitamin D which were, at least partly, published in the journal Frontiers in Endocrinology. The “Classic and Pleiotropic Actions of Vitamin D” research topic, edited in Frontiers in Endocrinology, also welcomed “insights from outside,” i.e., 4 topical papers submitted by authors who did not present their work at the EVIDAS 2017 conference. These articles have been labeled below as “invited papers.”

The article by Carol L. Wagner and Bruce W. Hollis (invited paper) reviewed on what is known about the roles of vitamin D status during pregnancy for both mother and the developing fetus (Wagner and Hollis). The recognized leaders in research on vitamin D supplementation during pregnancy reported that pregnant women receiving 4,000 IU/d of vitamin D3 throughout their pregnancy achieved 25-hydroxyvitamin D [25(OH)D] concentrations above 40 ng/ml (100 nmol/l) without any evidence of toxicity, as demonstrated by the maintenance of a normal calcaemia and no significant changes in 24-h urinary calcium excretion. The authors emphasized that benefits of the vitamin D supplementation program was associated with a reduced risk of preeclampsia and prematurity as well as other essential benefits, including better fetal neurodevelopmental parameters, improved lung maturation and respiratory function. This paper reviewed the results from several relevant clinical trials, and thereby highlighted that trial results should rather be analyzed with respect to achievable serum 25(OH)D concentrations, not just the vitamin D dose. Properly designed clinical trials have demonstrated—according to the published review—that higher maternal 25(OH)D concentrations reduced the risk of postnatal asthma, preeclampsia, preterm birth, and a variety of other gestational complications (Wagner and Hollis).

The trends toward changes in 25(OH)D levels and alterations of markers of calcium-phosphate metabolism were investigated over the 30 years (1981–2011) in a population of newborns and infants who were suspected of having a disorder in calcium-phosphate metabolism (*n* = 3163; mean age 9.0 ± 3.0 months) (Wójcik et al.). In neonates and infants, the 25(OH)D as low as < 10 ng/ml (< 25 nmol/l) was found in 4.5% of patients (*n* = 163), 10-20 ng/ml (25–50 nmol/l) was noted in 14.7% (*n* = 465), 20–30 ng/ml (50–75 nmol/l) in 23.9% (*n* = 756) and 30–50 ng/ml (75–125 nmol/l) in 35.9% (*n* = 1136). The mean 25(OH)D concentration was 37.5 ± 24.5 ng/ml (93.8 ± 61.2 nmol/l). In subjects with 25(OH)D concentration < 10 ng/ml (< 25 nmol/l), normal calcaemia (2.25–2.65 mmol/l) was found in 83.4% of neonates and infants (*n* = 136). Eighty-one subjects had 25(OH)D concentrations above 100 ng/ml (250 nmol/l), with co-existing serum calcium ranging from 2.6 mmol/l up to 4.38 mmol/l (mean Ca = 2.69 mmol/l). Hypocalcaemia (Ca < 2.25 mmol/l) was observed in 0.54% only. Of the total studied population, 13.8% of infants had calcium levels > 2.65 mmol/l (*n* = 435). In general, the average values of calcium-phosphate markers were within the age-adjusted reference range. The highest mean 25(OH)D concentration of 51.8 ng/ml ± 38.8 (129.5 ± 97.0 nmol/l) was noted in years 1981–1999 (*n* = 305). The lowest 25(OH)D value was observed in years 2010–2011 (29.0 ng/ml ± 13.6; 72.5 ± 34.0 nmol/l; *n* = 412). The trend to a decline in 25(OH)D concentration throughout the studied period appeared significant (*r* = -0.29, *p* < 0.0001) (Wójcik et al.).

Associations between vitamin D status and obesity have been extensively studied during the last decade, however, conflicting results have been reported. The carbohydrate and lipid metabolism parameters in overweight children and adolescents in relation to vitamin D insufficiency were analyzed by a research group from Russian Federation (Zakharova et al.). They confirmed a high proportion of vitamin D insufficiency in overweight and obese Russian children and adolescents (escalating along with the severity of obesity). In this comprehensive study, the authors reported on the relationships and interplay between the principal adipokines responsible for fat metabolism (leptin, adiponectin, resistin) and vitamin D metabolism (Zakharova et al.).

A complex endocrine pathology was reported in an invited paper from Ukraine, while the study pointed out essential vitamin D issues as a serious healthcare problem in Ukraine following the historical nuclear disaster in Chernobyl in 1980s (Komisarenko et al.). The 25(OH)D and markers of immune function in response to vitamin D intervention were investigated in adult patients with type 1 and type 2 diabetes mellitus (T1DM and T2DM, respectively) and coincident autoimmune thyroiditis (AIT). It was reported that patients with combined endocrine disorders (DM and AIT) with vitamin D deficiency/insufficiency had significantly increased concentrations of Th1-type cytokines and reduced concentrations of Th2-type cytokines (IL-4 and IL-5), IL-10, and IL-17. The results of the Ukrainian study showed that vitamin D3 supplementation in patients with T1DM and T2DM may reduce the activity of the inflammatory Th1-type cytokines, and increase the levels of Th2-type cytokines (Komisarenko et al.).

In a paper, authored by 35 contributors from Europe and North America, the rationale and plans for vitamin D food fortification were reviewed with a suggestions for action (Pilz et al.). The rationale for vitamin D food fortification includes a large proportion of worldwide populations with low 25(OH)D concentrations and the mounting evidence from observational and clinical trials, that higher 25(OH)D concentrations and vitamin D intake are associated with several non-skeletal health benefits such as reduced mortality rates, respiratory tract infections, asthma exacerbations and pregnancy complications. The results of a systematic voluntary food fortification program in Finland in 2003 were reviewed. Fat spreads and fluid milk products could be fortified. The mean overall increase in serum 25(OH)D concentration from 2000 to 2011 attributed to food fortification was 2.4 ng/ml (6 nmol/l). The suggested goal in this review was to bring everyone up to >20 ng/ml (>50 nmol/l). The mean dietary vitamin D intake in Finland in 2011 was 14 micrograms daily for men and 12 micrograms daily for women (10). As of April 30, 2019, this paper was viewed more than 11,000 times.

A study in Poland found that long-term acenocoumarol treatment due to recurrent venous thromboembolism, atrial fibrillation, or mechanical heart valve prostheses was significantly inversely correlated with serum 25(OH)D concentration (Sawicka-Powierza et al.). The authors of this interesting research concluded that acenocoumarol treatment, being today a largely used anticoagulation therapy, might be responsible for a decrease of 25(OH)D concentration. They acknowledged, however, that other reasons for the inverse correlation could not be ruled out (Sawicka-Powierza et al.).

Vitamin D supplementation guidelines were prepared for the general population and groups at risk of vitamin D deficiency in Poland by an expert panel of 28 vitamin D researchers, national specialist consultants and representatives of scientific societies (Rusinska et al.). In general, the guidelines recommended 800 to 2,000 IU/d vitamin D for those aged 11 to 75 years of age, lower doses for the younger, and higher ones for older or those at risk of vitamin D deficiency. However, about 15–30 min long sun exposure daily between 10 a.m. and 3 p.m. from May to September (depending on skin type, time of day and season) could provide some of the vitamin D, at least for the latitudes similar to Poland. Based on the available body of evidence cited in this review, the 25(OH)D of 30–50 ng/ml (75–125 nmol/l) was identified as the optimal concentration, with the potentially toxic concentrations >100 ng/ml (>250 nmol/l). The recommendations were based on beneficial skeletal and non-skeletal effects, derived largely from observational studies. The lack of a strong support from RCTs including vitamin D was attributed to trial design problems, in a way, and was discussed as a limitation in this guidance paper (Rusinska et al.). As of April 30, 2019, this paper had 33,261 total views.

The topic of vitamin D toxicity (VDT) was discussed from a clinical perspective by Marcinowska-Suchowierska et al.. The main hallmarks of VDT are hypercalciuria, hypercalcemia, suppressed parathyroid hormone, and 25(OH)D concentrations above 150 ng/ml (375 nmol/l). The most likely hypothesis to explain VDT is that high 25(OH)D concentrations saturate the vitamin D binding protein, thus increasing the bioavailability of 1,25(OH)2D for the target cell nucleus. In addition high concentrations of 25(OH)D can stimulate transcription through the VDR. The symptoms of VDT may include neuropsychiatric manifestations such as difficulty in concentration, confusion, depression, gastrointestinal symptoms such as vomiting, abdominal pain, constipation, cardiovascular manifestations such as hypertension, renal symptoms such as dehydration. Various aspects of VDT treatment were given in this article. The most common cause of VDT appears to be accidental overdosing of vitamin D due to a neglect, unawareness and/or manufacturing error (Marcinowska-Suchowierska).

A 12-week study in Gdansk, Poland, dealt with the role of vitamin D supplementation and exercise on blood cholesterol in elderly women (Prusik et al.). The study was conducted from the mid-October till mid-January. For those who were supplemented with vitamin D3 (4000 IU/d), no changes in lipid profile were observed. However, in those who were supplemented (mean serum 25(OH)D increased from 21 ng/ml to 38 ng/ml; from 52.5 nmol/ to 95 nmol/l) and did Nordic walking for about an hour three times per week, a decrease in total cholesterol, low-density lipoprotein cholesterol and triglycerides of about 10% was observed. Meanwhile in the control group, the high-density lipoprotein cholesterol increased (Prusik et al.).

An observational study (invited paper) found no relationship between maternal and cord blood vitamin D status and anthropometric measurements in term-born neonates at birth (Wierzejska et al.). A total of 94 mother-infant pairs were studied. On the other hand, a study in India of women with a mean serum 25(OH)D concentration of 18 ng/ml (45 nmol/l) at time of birth found that “fetal femur length and birth length were significantly shorter in mothers with low 25(OH)D (P < 0.01)” (11). A study in Finland involving 723 mother-child pairs concluded: “A sufficient maternal vitamin D status, specified as 25(OH)D above 50 nmol/L (20 ng/ml), may be a threshold above which the physiological requirements of pregnancy are achieved” (12).

A cross-sectional study of serum 25(OH)D concentrations in relation to activities of daily living (ADL) was conducted among octogenarians—residents of Vilnius city (Lithuania) from January 2017 to February 2018 (Alekna et al.). No association was found between seasonal variations of blood sampling and serum 25(OH)D concentration. Functional status of the oldest old subjects was evaluated based on bathing, dressing, toileting, transferring, continence, and feeding. Those in the lowest ADL category had 25(OH)D concentration near 7 ng/ml (17.5 nmol/l), those in the category 5: approx. 15 ng/ml (37.5 nmol/l), and those in the category 6: 20 ng/ml (50 nmol/l). The regression coefficient for 25(OH)D concentration vs. ADL category was 0.2 (*p* = 0.01). As highlighted by the authors, it was not possible in this study to determine whether ADL status was a cause or an effect of serum 25(OH)D concentration (Alekna et al.).

An interesting issue addressing the personalized individual response of the transcriptome on vitamin D supplementation was discussed by Carlberg. Before and 24 h after a vitamin D3 bolus, the chromatin and RNA were prepared from peripheral blood mononuclear cells for epigenome- and transcriptome-wide analysis. The study subjects showed a specific personalized response to vitamin D and could be stratified into high, mid and low responders. Comparable principles of vitamin D signaling were identified *in vivo* and *in vitro* concerning target gene responses as well as changes in chromatin accessibility (Carlberg).

A study conducted in Brazil (invited paper) found that the frequency of the Taql (C allele) vitamin D receptor was significantly lower in the controlled ovarian stimulation groups than in the control groups, and that follicle number, but not oocyte number, was lower in patients with Taql polymorphic (TC/CC) genotypes (Reginatto et al.). The same group also reported that the frequency of the Taql CC genotype was higher in polycystic ovary syndrome (PCOS) group, while the CT genotype was the most frequent in controls (13). These studies seem to underscore the importance of serum 25(OH)D concentration for reproductive health in humans, particularly among women intending to become pregnant.

Vitamin D receptor (VDR) gene polymorphic variants (ApaI rs7975232, BsmI rs1544410, TaqI rs731236, and Cdx2 rs11568820) were investigated by Belorussian and Lithuanian research group in the context of the risk of postmenopausal osteoporosis (PMO) (Marozik et al.). Patients with osteoporosis were three times more likely to carry the rs1544410 G/G genotype, when compared to controls. The rs7975232, rs1544410 and rs731236 variants were in a strong direct linkage disequilibrium (*P* < 0.0001), suggesting that risk alleles of these markers are preferably inherited jointly. For the bearers of C-G-C haplotype (consisting of rs7975232, rs1544410 and rs731236 unfavorable alleles), the risk of PMO was significantly higher (OR = 4.7, 95% CI 2.8 to 8.1, *p* < 0.0001) compared to controls. This haplotype appeared to be significantly over-represented in PMO group compared to all other haplotypes (Marozik et al.).

Further, the associations between vitamin D status and VDR gene polymorphisms were also investigated in the context of metabolic syndrome (MS) and its markers in middle-aged Russian women, and this was reported by Karonova et al.. The 25(OH)D) concentrations and four VDR gene polymorphisms rs1544410 (BsmI), rs7975232 (ApaI), rs731236 (TaqI) and rs2228570 (FokI) as well as metabolic syndrome (MS) parameters were investigated in 697 women aged between 30 to 55 years. Cases with vitamin D deficiency showed an increased risk of abdominal obesity (AO) [CI 95% 2.23; 1.15–4.30] and low high-density lipoprotein cholesterol (HDL-C) [CI95% 2.60; 1.04–6.49], compared to subjects with normal 25(OH)D concentration. An impaired glucose tolerance (IGT) and T2DM risk were present only when 25(OH)D concentration was < 39.0 nmol/l (15.6 ng/ml), however, the risk of MS did not differ between adequate vitamin D status subjects and the insufficient/deficient ones (p>0.05). T allele carriers (A) of rs7975232 had higher total cholesterol and low-density lipoprotein cholesterol levels compared with the GG (aa) genotypes. Similarly, GG (BB) genotype carriers of rs1544410 had higher triglyceride levels than subjects with A (b) allele carriers. However, VDR gene polymorphisms did not seem to be associated with an increased risk of MS. Karonova and colleagues concluded that vitamin D deficiency, rs7975232 and rs1544410 VDR gene variants were associated with MS parameters in Russian middle-aged women (Karonova et al.).

Shymanskyi et al. conducted studies in female Wistar rats evaluating the effect of vitamin D in improving glucocorticoid-induced changes in bone marrow cells related to bone remodeling (Shymanskyi et al.). They observed that prednisolone-induced abnormalities in glucocorticoid and RANKL/RANK/OPG signaling pathways were associated with the impairments of vitamin D auto/paracrine system in bone marrow cells, and could be ameliorated by vitamin D supplementation (Shymanskyi et al.).

It is well-documented that secondary hyperparathyroidism is a major complication affecting bone metabolism in patients with chronic kidney disease. Various strategies have been developed to prevent and treat secondary hyperparathyroidism. One of the management strategies is to provide patients with 1,25-dihydroxyvitamin D3 or one of its active analogs with, in order to enhance intestinal calcium absorption and to decrease the expression and production of parathyroid hormone (PTH). The other strategy is to use a calcimimetic that is recognized by the calcium sensor resulting in a decrease in the signal that stimulates PTH expression and production. Zawierucha et al. reported a study whereby they treated 131 patients with hemodialysis and uncontrolled PTH secretion for 12 months, using intravenous paricalcitol, oral cinacalcet or a combination of both. Interestingly, they demonstrated that intravenous paricalcitol had a significant effect on the reduction of PTH activity whereas the combination of paricalcitol and cinacalcet had no additional benefit.

## Summary

A large-scale debate about non-calcemic action and the extra-skeletal health benefits of vitamin D continues, although limitations in vitamin D research and study design, vitamin D controversies and negative results are also extensively discussed in the current literature. The current research topic in Frontiers in Endocrinology may actually cover the needs, and may provide important input into the content of a well-managed scientific exchange of views. The series of the international conference “Vitamin D—minimum, maximum, optimum” (EVIDAS) provide a unique forum targeting basic scientists, clinical researchers, physicians and other health care professionals to discuss these dilemmas, controversies, pros and cons, as well as to evaluate most recent advances and updated information regarding vitamin D and health. The 4th International “Vitamin D- minimum, maximum, optimum” (EVIDAS 2019) conference which is scheduled in Warsaw, Poland, for October 11-12, 2019 will follow its basic concept to offer an opportunity for scientists and clinicians to present new developments, updated and critical information on the physiology, pathophysiology and health-related issues of vitamin D.

## Author Contributions

All authors listed have made a substantial, direct and intellectual contribution to the work, and approved it for publication.

### Conflict of Interest Statement

The authors declare that the research was conducted in the absence of any commercial or financial relationships that could be construed as a potential conflict of interest.
